# Supplementary material for: ProteinShader: illustrative rendering of macromolecules
Source: BMC Struct Biol. 2009 Mar 30;9:19. doi: 10.1186/1472-6807-9-19 (PMC2672931; doi:10.1186/1472-6807-9-19)
Supplement: Additional file 1 — ProteinShader program without source code. This compressed file contains the complete ProteinShader program including associated libraries, but no source code. A README.txt file gives an overview of the ProteinShader distribution, and the index.html file in the help subdirectory has directions on getting started with the program as well as a set of tutorials. [file 1472-6807-9-19-S1.zip › ProteinShader-beta-0_9_4-binary/help/api/org/proteinshader/gui/components/controlpanel/CartoonVisibilityPanel.html]

CartoonVisibilityPanel (ProteinShader API)


|  |  |  |
| --- | --- | --- |
| **Overview** \| **Package** \| **Class** \| **Use** \| **Tree** \| **Deprecated** \| **Index** \| **Help** | |  |
| **PREV CLASS**   **NEXT CLASS** | **FRAMES**    **NO FRAMES**     **All Classes** |  |
| SUMMARY: NESTED \| FIELD \| CONSTR \| METHOD | DETAIL: FIELD \| CONSTR \| METHOD |  |


---


## org.proteinshader.gui.components.controlpanel Class CartoonVisibilityPanel

```
java.lang.Object
  java.awt.Component
      java.awt.Container
          javax.swing.JComponent
              javax.swing.JPanel
                  org.proteinshader.gui.components.controlpanel.VisibilityPanel
                      org.proteinshader.gui.components.controlpanel.CartoonVisibilityPanel
```

**All Implemented Interfaces:**: ImageObserver, MenuContainer, Serializable, Accessible

---

``` public class CartoonVisibilityPanel extends VisibilityPanel ```

This control panel allows the user to modify the visibility status and
alpha value of Segments.

**See Also:**: Serialized Form

---

**Nested Class Summary**

**Nested classes/interfaces inherited from class javax.swing.JPanel**

`JPanel.AccessibleJPanel`

**Nested classes/interfaces inherited from class javax.swing.JComponent**

`JComponent.AccessibleJComponent`

**Nested classes/interfaces inherited from class java.awt.Container**

`Container.AccessibleAWTContainer`

**Nested classes/interfaces inherited from class java.awt.Component**

`Component.AccessibleAWTComponent, Component.BltBufferStrategy, Component.FlipBufferStrategy`


| **Field Summary** | |
| --- | --- |
| `static String` | `MENU_NAME`             The menu name "Cartoon Visibility" will be returned by the toString() method. |

**Fields inherited from class org.proteinshader.gui.components.controlpanel.VisibilityPanel**

`SLIDER_INIT`

**Fields inherited from class javax.swing.JComponent**

`accessibleContext, listenerList, TOOL_TIP_TEXT_KEY, ui, UNDEFINED_CONDITION, WHEN_ANCESTOR_OF_FOCUSED_COMPONENT, WHEN_FOCUSED, WHEN_IN_FOCUSED_WINDOW`

**Fields inherited from class java.awt.Component**

`BOTTOM_ALIGNMENT, CENTER_ALIGNMENT, LEFT_ALIGNMENT, RIGHT_ALIGNMENT, TOP_ALIGNMENT`

**Fields inherited from interface java.awt.image.ImageObserver**

`ABORT, ALLBITS, ERROR, FRAMEBITS, HEIGHT, PROPERTIES, SOMEBITS, WIDTH`


**Constructor Summary**

`CartoonVisibilityPanel(Mediator mediator,
Frame dialogOwner,
RadioPanel radioPanel)`
  
          Constructs a CartoonVisibilityPanel.


| **Method Summary** | |
| --- | --- |
| `void` | `modifyGlobal(VisibilityEnum visibility, float alpha)`             This helper method for applyVisibility() sets up the the SegmentModifier and then calls on modifyGlobal() method of the radio panel. |
| `void` | `modifyHelices(VisibilityEnum visibility, float alpha)`             This helper method for applyVisibility() sets up a SegmentModifier and then calls on modifyHelices() method of the radio panel. |
| `void` | `modifyLoops(VisibilityEnum visibility, float alpha)`             This helper method for applyVisibility() sets up a SegmentModifier and then calls on the modifyLoops() method of the radio panel. |
| `void` | `modifySelected(VisibilityEnum visibility, float alpha)`             This helper method for applyVisibility() sets up the SegmentModifier and then calls on the modifySelected() method of the radio panel. |
| `void` | `modifyStrands(VisibilityEnum visibility, float alpha)`             This helper method for applyVisibility() sets up a SegmentModifier and then calls on the modifyStrands() method of the radio panel. |
| `String` | `toString()`             Returns a name suitable for use in a menu. |

**Methods inherited from class org.proteinshader.gui.components.controlpanel.VisibilityPanel**

`applyVisibility, getPercentField, getSliderValue, modifyGlobal, modifyHelices, modifyLoops, modifySelected, modifyStrands, setPercentField, setSliderValue`

**Methods inherited from class javax.swing.JPanel**

`getAccessibleContext, getUI, getUIClassID, paramString, setUI, updateUI`

**Methods inherited from class javax.swing.JComponent**

`addAncestorListener, addNotify, addVetoableChangeListener, computeVisibleRect, contains, createToolTip, disable, enable, firePropertyChange, firePropertyChange, firePropertyChange, fireVetoableChange, getActionForKeyStroke, getActionMap, getAlignmentX, getAlignmentY, getAncestorListeners, getAutoscrolls, getBorder, getBounds, getClientProperty, getComponentGraphics, getComponentPopupMenu, getConditionForKeyStroke, getDebugGraphicsOptions, getDefaultLocale, getFontMetrics, getGraphics, getHeight, getInheritsPopupMenu, getInputMap, getInputMap, getInputVerifier, getInsets, getInsets, getListeners, getLocation, getMaximumSize, getMinimumSize, getNextFocusableComponent, getPopupLocation, getPreferredSize, getRegisteredKeyStrokes, getRootPane, getSize, getToolTipLocation, getToolTipText, getToolTipText, getTopLevelAncestor, getTransferHandler, getVerifyInputWhenFocusTarget, getVetoableChangeListeners, getVisibleRect, getWidth, getX, getY, grabFocus, isDoubleBuffered, isLightweightComponent, isManagingFocus, isOpaque, isOptimizedDrawingEnabled, isPaintingTile, isRequestFocusEnabled, isValidateRoot, paint, paintBorder, paintChildren, paintComponent, paintImmediately, paintImmediately, print, printAll, printBorder, printChildren, printComponent, processComponentKeyEvent, processKeyBinding, processKeyEvent, processMouseEvent, processMouseMotionEvent, putClientProperty, registerKeyboardAction, registerKeyboardAction, removeAncestorListener, removeNotify, removeVetoableChangeListener, repaint, repaint, requestDefaultFocus, requestFocus, requestFocus, requestFocusInWindow, requestFocusInWindow, resetKeyboardActions, reshape, revalidate, scrollRectToVisible, setActionMap, setAlignmentX, setAlignmentY, setAutoscrolls, setBackground, setBorder, setComponentPopupMenu, setDebugGraphicsOptions, setDefaultLocale, setDoubleBuffered, setEnabled, setFocusTraversalKeys, setFont, setForeground, setInheritsPopupMenu, setInputMap, setInputVerifier, setMaximumSize, setMinimumSize, setNextFocusableComponent, setOpaque, setPreferredSize, setRequestFocusEnabled, setToolTipText, setTransferHandler, setUI, setVerifyInputWhenFocusTarget, setVisible, unregisterKeyboardAction, update`

**Methods inherited from class java.awt.Container**

`add, add, add, add, add, addContainerListener, addImpl, addPropertyChangeListener, addPropertyChangeListener, applyComponentOrientation, areFocusTraversalKeysSet, countComponents, deliverEvent, doLayout, findComponentAt, findComponentAt, getComponent, getComponentAt, getComponentAt, getComponentCount, getComponents, getComponentZOrder, getContainerListeners, getFocusTraversalKeys, getFocusTraversalPolicy, getLayout, getMousePosition, insets, invalidate, isAncestorOf, isFocusCycleRoot, isFocusCycleRoot, isFocusTraversalPolicyProvider, isFocusTraversalPolicySet, layout, list, list, locate, minimumSize, paintComponents, preferredSize, printComponents, processContainerEvent, processEvent, remove, remove, removeAll, removeContainerListener, setComponentZOrder, setFocusCycleRoot, setFocusTraversalPolicy, setFocusTraversalPolicyProvider, setLayout, transferFocusBackward, transferFocusDownCycle, validate, validateTree`

**Methods inherited from class java.awt.Component**

`action, add, addComponentListener, addFocusListener, addHierarchyBoundsListener, addHierarchyListener, addInputMethodListener, addKeyListener, addMouseListener, addMouseMotionListener, addMouseWheelListener, bounds, checkImage, checkImage, coalesceEvents, contains, createImage, createImage, createVolatileImage, createVolatileImage, disableEvents, dispatchEvent, enable, enableEvents, enableInputMethods, firePropertyChange, firePropertyChange, firePropertyChange, firePropertyChange, firePropertyChange, firePropertyChange, getBackground, getBounds, getColorModel, getComponentListeners, getComponentOrientation, getCursor, getDropTarget, getFocusCycleRootAncestor, getFocusListeners, getFocusTraversalKeysEnabled, getFont, getForeground, getGraphicsConfiguration, getHierarchyBoundsListeners, getHierarchyListeners, getIgnoreRepaint, getInputContext, getInputMethodListeners, getInputMethodRequests, getKeyListeners, getLocale, getLocation, getLocationOnScreen, getMouseListeners, getMouseMotionListeners, getMousePosition, getMouseWheelListeners, getName, getParent, getPeer, getPropertyChangeListeners, getPropertyChangeListeners, getSize, getToolkit, getTreeLock, gotFocus, handleEvent, hasFocus, hide, imageUpdate, inside, isBackgroundSet, isCursorSet, isDisplayable, isEnabled, isFocusable, isFocusOwner, isFocusTraversable, isFontSet, isForegroundSet, isLightweight, isMaximumSizeSet, isMinimumSizeSet, isPreferredSizeSet, isShowing, isValid, isVisible, keyDown, keyUp, list, list, list, location, lostFocus, mouseDown, mouseDrag, mouseEnter, mouseExit, mouseMove, mouseUp, move, nextFocus, paintAll, postEvent, prepareImage, prepareImage, processComponentEvent, processFocusEvent, processHierarchyBoundsEvent, processHierarchyEvent, processInputMethodEvent, processMouseWheelEvent, remove, removeComponentListener, removeFocusListener, removeHierarchyBoundsListener, removeHierarchyListener, removeInputMethodListener, removeKeyListener, removeMouseListener, removeMouseMotionListener, removeMouseWheelListener, removePropertyChangeListener, removePropertyChangeListener, repaint, repaint, repaint, resize, resize, setBounds, setBounds, setComponentOrientation, setCursor, setDropTarget, setFocusable, setFocusTraversalKeysEnabled, setIgnoreRepaint, setLocale, setLocation, setLocation, setName, setSize, setSize, show, show, size, transferFocus, transferFocusUpCycle`

**Methods inherited from class java.lang.Object**

`clone, equals, finalize, getClass, hashCode, notify, notifyAll, wait, wait, wait`

**Field Detail**

### MENU\_NAME

```
public static final String MENU_NAME
```

:   The menu name "Cartoon Visibility" will be returned by
    the toString() method.

    **See Also:**: Constant Field Values


**Constructor Detail**

### CartoonVisibilityPanel

```
public CartoonVisibilityPanel(Mediator mediator,
                              Frame dialogOwner,
                              RadioPanel radioPanel)
```

:   Constructs a CartoonVisibilityPanel.

    **Parameters:**: `mediator` - the centralized Mediator that most listeners need to call on to accomplish their task.: `dialogOwner` - the owner of any Dialogs opened from the SelectorPanel or null if there is no owner).: `radioPanel` - the common RadioPanel used by subpanels.


**Method Detail**

### modifySelected

```
public void modifySelected(VisibilityEnum visibility,
                           float alpha)
                    throws ColorOutOfRangeException
```

:   This helper method for applyVisibility() sets up the
    SegmentModifier and then calls on the modifySelected() method
    of the radio panel.

    :   **Specified by:**: `modifySelected` in class `VisibilityPanel`
    :   **Parameters:**: `visibility` - the visibility state.: `alpha` - the alpha value associated with the RGB color. **Throws:**: `ColorOutOfRangeException` - if alpha is less than 0.0 or greater than 1.0.

---


### modifyHelices

```
public void modifyHelices(VisibilityEnum visibility,
                          float alpha)
                   throws ColorOutOfRangeException
```

:   This helper method for applyVisibility() sets up a SegmentModifier
    and then calls on modifyHelices() method of the radio panel.

    :   **Specified by:**: `modifyHelices` in class `VisibilityPanel`
    :   **Parameters:**: `visibility` - the visibility state.: `alpha` - the alpha value associated with the RGB color. **Throws:**: `ColorOutOfRangeException` - if alpha is less than 0.0 or greater than 1.0.

---


### modifyStrands

```
public void modifyStrands(VisibilityEnum visibility,
                          float alpha)
                   throws ColorOutOfRangeException
```

:   This helper method for applyVisibility() sets up a SegmentModifier
    and then calls on the modifyStrands() method of the radio panel.

    :   **Specified by:**: `modifyStrands` in class `VisibilityPanel`
    :   **Parameters:**: `visibility` - the visibility state.: `alpha` - the alpha value associated with the RGB color. **Throws:**: `ColorOutOfRangeException` - if alpha is less than 0.0 or greater than 1.0.

---


### modifyLoops

```
public void modifyLoops(VisibilityEnum visibility,
                        float alpha)
                 throws ColorOutOfRangeException
```

:   This helper method for applyVisibility() sets up a SegmentModifier
    and then calls on the modifyLoops() method of the radio panel.

    :   **Specified by:**: `modifyLoops` in class `VisibilityPanel`
    :   **Parameters:**: `visibility` - the visibility state.: `alpha` - the alpha value associated with the RGB color. **Throws:**: `ColorOutOfRangeException` - if alpha is less than 0.0 or greater than 1.0.

---


### modifyGlobal

```
public void modifyGlobal(VisibilityEnum visibility,
                         float alpha)
                  throws ColorOutOfRangeException
```

:   This helper method for applyVisibility() sets up the the
    SegmentModifier and then calls on modifyGlobal() method of the
    radio panel.

    :   **Specified by:**: `modifyGlobal` in class `VisibilityPanel`
    :   **Parameters:**: `visibility` - the visibility state.: `alpha` - the alpha value associated with the RGB color. **Throws:**: `ColorOutOfRangeException` - if alpha is less than 0.0 or greater than 1.0.

---


### toString

```
public String toString()
```

:   Returns a name suitable for use in a menu.

    :   **Overrides:**: `toString` in class `Component`
    :   **Returns:**: The menu name as a String.


---


|  |  |  |
| --- | --- | --- |
| **Overview** \| **Package** \| **Class** \| **Use** \| **Tree** \| **Deprecated** \| **Index** \| **Help** | |  |
| **PREV CLASS**   **NEXT CLASS** | **FRAMES**    **NO FRAMES**     **All Classes** |  |
| SUMMARY: NESTED \| FIELD \| CONSTR \| METHOD | DETAIL: FIELD \| CONSTR \| METHOD |  |


---

# *Copyright © 2007-2008*
